# Molecular Detection of *Bartonella* spp. and Hematological Evaluation in Domestic Cats and Dogs from Bangkok, Thailand

**DOI:** 10.3390/pathogens10050503

**Published:** 2021-04-22

**Authors:** Phirabhat Saengsawang, Gunn Kaewmongkol, Tawin Inpankaew

**Affiliations:** 1 Center for Agricultural Biotechnology, Kamphaeng Saen Campus, Kasetsart University, Nakhon Pathom 73140, Thailand; phirabhat.s@gmail.com; 2 Center of Excellence on Agricultural Biotechnology (AG-BIO/PERDO-CHE), Bangkok 10900, Thailand; 3 Akkhraratchakumari Veterinary College, Walailak University, Nakhon Si Thammarat 80161, Thailand; 4 Department of Companion Animal Clinical Sciences, Faculty of Veterinary Medicine, Kasetsart University, Bangkok 10900, Thailand; gunn_kaew@yahoo.com; 5 Department of Parasitology, Faculty of Veterinary Medicine, Kasetsart University, Bangkok 10900, Thailand

**Keywords:** *Bartonella*, dog, cat, phylogenetic, hematology, Thailand

## Abstract

(1) Background: *Bartonella* spp. are Gram-negative, facultative, intracellular bacteria transmitted by hematophagous insects. Several species cause zoonotic diseases such as cat-scratch disease. *Bartonella henselae* and *Bartonella clarridgeiae* are the main species found in Thailand, however, there have been few studies on *Bartonella* spp. In addition, the hematological evaluation of *Bartonella*-infected animals is limited in Thailand. The aims of this study were prevalence investigation and hematological evaluation of *Bartonella*-infected dogs and cats residing in Bangkok, Thailand. (2) Methods: In total, 295 dogs and 513 cats were molecularly evaluated to detect *Bartonella* spp. using PCR with primers targeting the partial *gltA*, *rpoB*, *ftsZ*, *ribC*, and *groEL* genes. In total, 651 domestic animals were evaluated for hematological parameters compared between *Bartonella*-positive and *Bartonella*-negative animals. (3) Results: Overall, the prevalence of *Bartonella* spp. was 1.61% which was found only in free-ranging cats (2.83%). *Bartonella henselae* and *B. clarridgeiae* were confirmed from a concatenated phylogenetic tree of partial *gltA* and *ribC* genes, with 100% bootstrapping replication. For other housekeeping gene sequences, mixed infection was expected from the amplicons of *rpoB*, *ftsZ*, and *groEL*. Importantly, the mean corpuscular volume (MCV) was significantly increased in *Bartonella*-positive cats. (4) Conclusions: We suggest that *B. henselae* and *B. clarridgeiae* are important species and are still circulating in domestic animals, especially cats. The evaluation of blood parameters, especially a raised MCV, should be of concern in *Bartonella* infection in asymptomatic cats. Additionally, the knowledge of how to prevent *Bartonella*-related diseases should be promoted with people in at-risk situations.

## 1. Introduction

*Bartonella* spp. are Gram-negative, facultative, fastidious intracellular bacteria causing various pathological changes and bacteremia in their natural and accidental hosts. Several bloodsucking arthropods have been confirmed as vectors transmitting these pathogens including cat fleas (*Ctenocephalides felis*) [1], human fleas (*Pulex irritans*) [2], human lice (*Pediculus humanus*) [3], and sandflies (*Luztomyia verrucarum*) [4]. Additionally, some tick species are suggested as possible vectors of *Bartonella* spp. such as the brown dog tick (*Rhipicephalus sanguineus*) [5] and the Ixodid tick (*Ixodes* spp. [6] and *Dermacentor* spp. [7]). Up to the present, a number of new species of *Bartonella* and candidatus Bartonella have been described and more than 35 species have revealed their whole genome.

Human bartonelloses are caused by *B. henselae* (cat-scratch disease), *B. bacilliformis* (Carrion’s disease and verruga peruana), and *B. quintana* (trench fever) [8]. Humans play a role as accidental hosts being infected by *Bartonella* spp. from various animal hosts, both companion and wild animals [9]. Bartonellosis mainly affects immunocompromised patients, however, only regional lymphadenopathy occurs in immunocompetent people [10]. At least 13 species are considered as zoonotic agents and the most zoonotic-prevalent species is *B. henselae*, which can cause an asymptomatic infection among the hosts, such as carnivores, rodents, and ruminants [10,11,12].

Dogs and cats are defined as family members sharing household environments with humans in both urban and rural areas, and they are considered as a source of zoonotic infection, especially of bartonellosis [13]. In addition, dogs and cats are major mammals that can act as reservoirs of *Bartonella* spp. [14] and are defined as the main animals transmitting these pathogens to humans. Importantly, cats have been described as a primary reservoir animal of human bartonellosis [15], but dogs are defined as accidental hosts of these pathogens due to a lack of clear evidence [16]. The clinical signs in cats are mostly mild compared with dogs, however, some hematological parameters of both animals are changed [14]. The evaluation of blood parameters in *Bartonella*-infected animals, particularly cats and dogs, is still unclear. Several studies in other countries have evaluated the blood parameters in *Bartonella*-infected animals. Importantly, blood parameters are frequently used to diagnose and monitor animal health. Even if domestic cats and dogs are popular in Thailand, there has been little hematological study comparing uninfected- and infected-*Bartonella* animals.

In Thailand, dogs and cats that are both free-ranging and owned cohabit human communities. Bangkok is a province where the density of temples and household are considered high. Free-ranging animals were frequently found in temples which were a place for cultural activities and festivals, and owned animals were also found in the household. Significantly, the pattern of familiarity between humans and their belonging animals might induce zoonotic infection, particularly bartonellosis. The main proven modes of transmission in the human are animal scratch or bite with *Bartonella*-contaminated vector feces, and blood-sucking arthropod bite [14]. However, there have been few studies reporting evidence of *Bartonella* in domestic animals in Thailand. The objectives of this study were to investigate the prevalence of *Bartonella* spp. in domestic dogs and cats and to evaluate the hematological parameters of *Bartonella*-infected dogs and cats in Bangkok, Thailand.

## 2. Results

### 2.1. Animal Demographic Characteristics

In total, 808 blood samples were collected from domestic cats (*n* = 513) and dogs (*n* = 295) residing in Bangkok, Thailand, consisting of 634 free-ranging (from temples) and 174 owned animals (from KUVTH). The ratio of males to females in the animals was 1.26:1 in the owned and 0.83:1 in the free-ranging animals. The mean ± standard deviation age of studied animals was 3.03 ± 2.01 years in the free-ranging animals (2.83 ± 1.71 years in cats and 3.29 ± 2.33 years in dogs), and in the owned animals was 3.47 ± 1.75 years (3.22 ± 1.71 years in cats and 3.58 ± 1.77 years in dogs). The numbers for related factors (gender, living location, breed, animal species, age group, and ectoparasite infestation) are presented in Table 1.

### 2.2. Prevalence of Bartonella spp.

**Figure 1:**
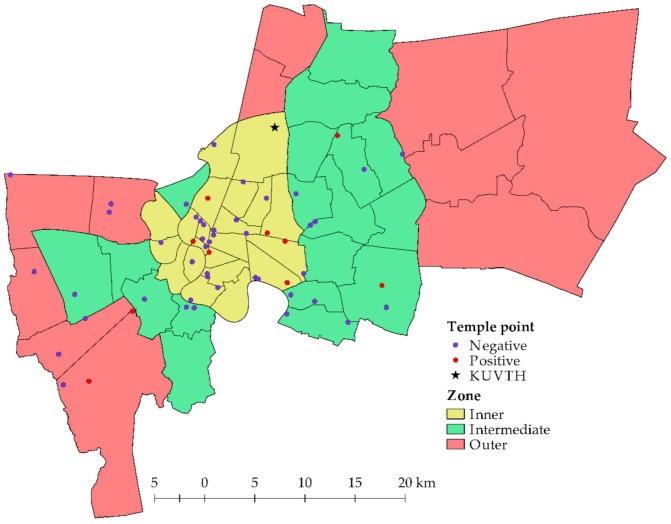
Map of Bangkok and studied temples; each polygon indicates a district boundary; each circular symbol indicates a temple location; and the asterisk symbol indicates the location of KUVTH.

The overall prevalence of *Bartonella* spp. in the domestic dogs and cats was 1.61% (13/808). Only domestic cats presented *Bartonella* DNA in extracted blood samples, however, there was an evidence only in free-ranging cats (2.83%) residing in temples (Table 2). Notably, there was no *Bartonella*-positive blood in domestic dogs (both free-ranging and owned animals) in the current study. *Bartonella* spp. were detected in animals residing in 10/54 (18.52%) temple clusters that were located in three zones of Bangkok (Figure 1). The positive proportions in the separate zones were in the range of 10.53–23.08% (6/26 clusters in the inner zone, 2/19 clusters in the intermediate zone, 2/9 clusters in the outer zone) and there were no significant differences between zones (*p* = 0.35). Additionally, there were no significant differences for gender (*p* = 0.34), age group (*p* = 0.51), and ectoparasite infestation (*p* = 0.22) in the examined animals.

### 2.3. Phylogenetic Analysis

**Figure 2:**
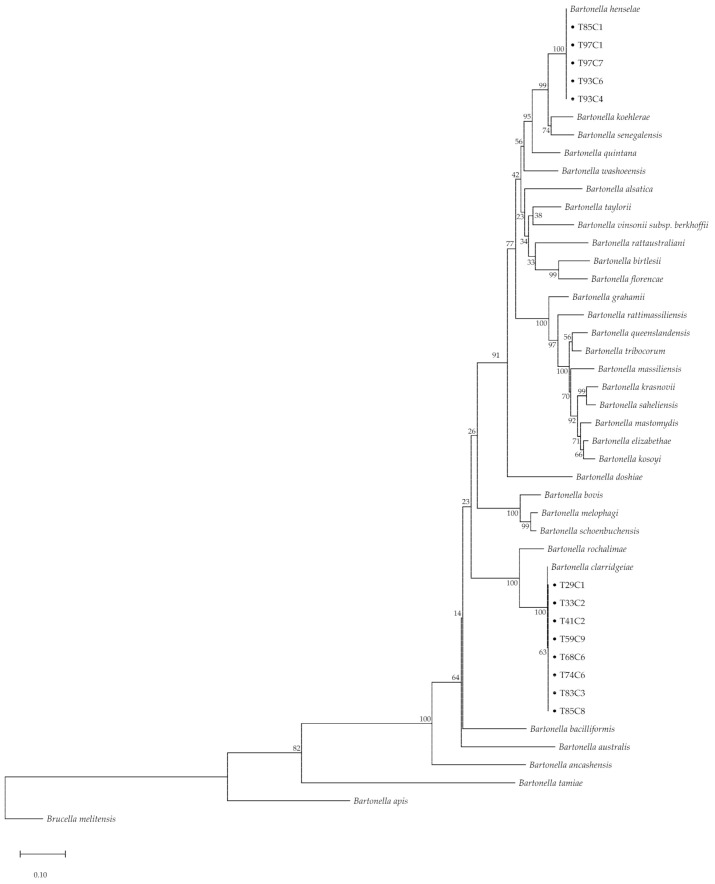
Phylogenetic tree of concatenated sequences (*gltA* and *ribC*) of *Bartonella*-positive cats (maximum likelihood tree with GTR + G model with 500 bootstrapping replications); *Brucella melitensis* is provided as an outgroup; and dots indicate positive samples from the current study.

The BLAST results of all *gltA*-positive amplicons showed a similar direction with matched species of *ribC* gene results. However, *rpoB*, *ftsZ*, and *groEL* revealed different species (Table 3). In addition, the amplicons of five samples (38.46%) showed two species of *Bartonella*. Amplicons of *gltA* and *ribC* matched with two species of *Bartonella* consisting of *B. henselae* (%identity = 97.01–100% of *gltA* and 86.62–100% of *ribC*) and *B. clarridgeiae* (%identity = 94.59–100% of *gltA* and 99.54–100% of *ribC*). Additionally, the similarity was up to 100% for *rpoB*, *ftsZ,* and *groEL* in all sequences, and details of sequencing data are revealed in Supplementary Table S1. The concatenated phylogenetic tree based on the maximum likelihood (ML) method with the general time reversible (GTR) model and a Gamma distribution based on partial *gltA* and *ribC* sequences showed two lineages of *Bartonella* that were identical to *B. henselae* and *B. clarridgeiae*, with 100% bootstrapping replication (Figure 2).

### 2.4. Hematological Comparison

**Figure 3:**
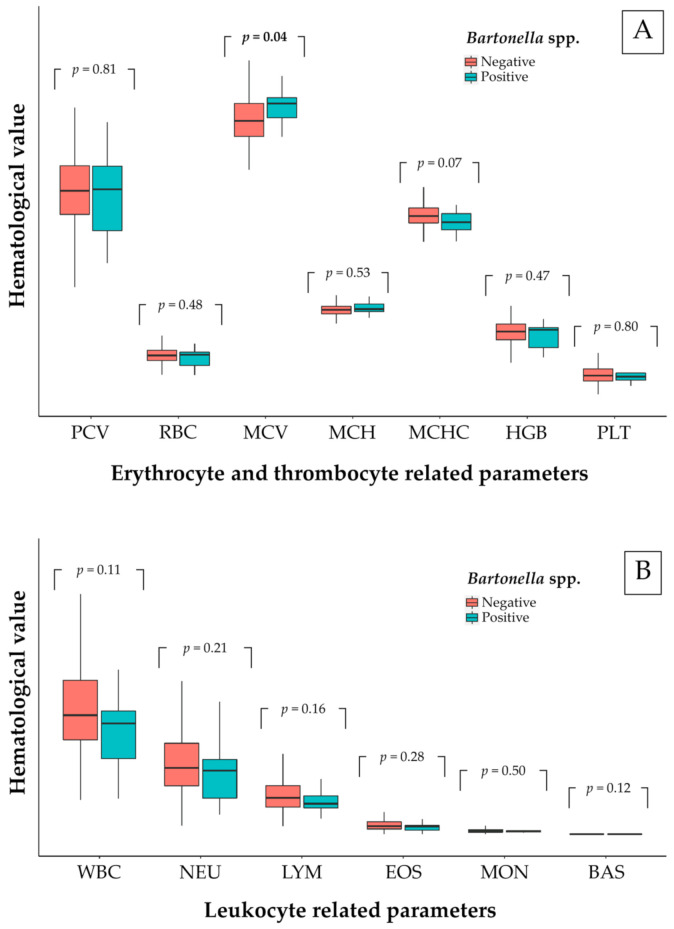
Box plot of each related hematological parameter in free-ranging cats; (**A**) erythrocytes and thrombocytes and (**B**) leukocytes.

In total, 650 examined animals (80.45%) were evaluated for blood parameters. The hematological results of the examined animal population were presented in Table 4. The hematological evaluation was carried out in *Bartonella*-positive animal groups and so there was only a comparison made for free-ranging cats. The hematological comparison between positive and negative *Bartonella* spp. of free-ranging cats is shown in Figure 3. There were no significant differences in blood parameters between *Bartonella*-positive and *Bartonella*-negative animals, except for MCV of erythrocytes. There was a significantly raised value for MCV (48.65 ± 4.78 fL for non-infected and 51.02 ± 3.60 fL for infected free-ranging cats) in *Bartonella*-infected free-ranging cats (*p* < 0.05). Moreover, more than 36% of positive cats had low PCV, HGB, and MCHC. The reference of blood parameters was included in Supplementary Table S2.

## 3. Discussion

This study detected evidence of *Bartonella* spp. in domestic dogs and cats in Bangkok, Thailand. The results indicated that the prevalence of *Bartonella* in the total cat population evaluated was 2.53%, however, *Bartonella* spp. was observed only in free-ranging cats. The current study found two species of *Bartonella* (*B. henselae* and *B. clarridgeiae*) which was similar with another study in Thailand that also reported that *B. henselae* and *B. clarridgeiae* as the main species in this region [17]. Evidence of *Bartonella* bacteremia was found in 13 cats, however, all dogs were negative to *Bartonella* in this study. In cats, this positive proportion contrasted with other *Bartonella* prevalence reported in Brazil [18,19], Greece [20], Italy [21,22], Germany [23], New Caledonia [24], USA [25,26,27,28,29], Saudi Arabia [30], Israel [31], Portugal [32], France [33,34], and Thailand [17], but was significantly similar with studies in Italy [35,36], Spain [37], Scotland [38], Ireland [39], and China [13]. Moreover, our study also demonstrated that the prevalence of *Bartonella* spp. in dogs was not significantly different from studies in China [13], Brazil [18], Spain [37], Cape Verde [40], and Grenada [41], but contrasted with other studies where *Bartonella* spp. were detected in cardiac tissues [42,43] and blood [42,44,45,46]. Importantly, there was a chance of an event that *Bartonella* was positive only in cardiac tissue but no evidence in blood [42]. Conversely, the detection in dogs in the current study was different from another study conducted in Thailand [46]. Additionally, dogs were defined as accidental hosts with some cardiac abnormalities [47] and cats were the major reservoir hosts presenting only subclinical disease or non-specific signs [48]. This explained why dogs were seldom detected as having *Bartonella* DNA in their blood samples. Comparisons of prevalence differences between recent and previous studies are presented in Supplementary Table S3. This different prevalence was affected from several different factors mainly including the detection technique, studied region, and host type. The geographic location and host type were considered as factors related to the prevalence of *Bartonella* [49]. Strayed animals frequently revealed a higher prevalence of *Bartonella* than owned animals [49] and these were caused by the difference of caring pattern, ectoparasite control, and ranging area. Thus, the negative prevalence in owned animals in this study was also caused by these aspects. Importantly, cyclic bacteremia of *Bartonella* was the main factor causing a false negative. For the detection method, sensitivity was the impacted factor that caused the different prevalence. Moreover, the concentration of *Bartonella* DNA might not match with the sensitivity of the test [47] and caused the different prevalence rate. The real-time and nested PCR presented a higher sensitivity than conventional PCR for *Bartonella* detection [13]. However, cPCR was less time and cost consumption than others. Additionally, various primers for cPCR had been completely produced compared to the real-time and nested PCR.

The prevalence of *Bartonella* spp. in the current study is quite low. The low prevalence in this study was suspected to be caused by direct pathogen detection from the bacteremia level in blood samples [44,50,51] and cyclic bacteremia characteristic of *Bartonella* spp. [52] at the sampling time. Additionally, some studies mentioned the lack of PCR sensitivity for *Bartonella* spp. detection in clinical samples, e.g., blood, other body fluids, and tissues [53]. Compared with the bacterial culture, PCR was suggested as a highly successful technique for *Bartonella* diagnosis in humans and experimental cats [54]. Although nested PCR was more sensitive than cPCR, the available primers for cPCR that used to target *Bartonella* spp. were quite more abundant. Due to the phylogenetic study, the longest sequence product of cPCR was more useful than the nested PCR for analysis. Additionally, the *Bartonella Alphaproteobacteria* growth medium (BAPGM) pre-enrichment was suggested for the specimen culture in *Bartonella* spp. to increase the sensitivity rate of PCR detection compared with the direct DNA extraction from pure samples [46]. Even if BAPGM increased the PCR sensitivity, non-*Bartonella* spp. had been also co-isolated with *Bartonella* spp. especially environmental bacteria, non-pathogenic commensal organism, and skin normal flora [55,56], and contamination was considered.

Only free-ranging cats were found with *Bartonella* DNA. Additionally, some studies concluded that the prevalence of *Bartonella* infection in stray animals was higher than in pet animals [16,49]. However, the current study indicated no associated factor that was significantly related with *Bartonella*-positive animals. This was consistent with other studies that found no difference in prevalence based on gender, residing location, living pattern, age, and ectoparasitic appearance [21]. A contrasting result in Thailand reported that young cats and flea-infested cats were mostly found with *Bartonella* spp [17]. We found a high positive proportion in the inner zone of Bangkok where there are many residences on crown land. This area should be considered for the introduction of disease transmission protection against zoonotic diseases, especially bartonellosis.

*Bartonella henselae* and *B. clarridgeiae* were the major species detected in cats [16]. These species have been reported in various countries in Southeast Asia [57,58,59,60]. In previous reports, Thailand had endocarditis patients infected with *B. henselae* [61] and *B. vinsonii* subsp. *arupensis* [62]. Of these, there was evidence to support the impact of zoonotic *Bartonella* infection in Thailand. *Bartonella henselae* was reported as the main species causing zoonotic infection, especially via cat-scratch disease. In Southeast Asia, it has been suggested that *B. henselae* and *B. clarridgeiae* are the species affecting human health and of veterinary importance [63]. In the main, *B. henselae* and *B. clarridgeiae* are transmitted to humans via cat fleas (*C. felis*) [64] which are generally found on cats and dogs worldwide [65] and *C. felis* is the major vector as the pathogen-transmissible insect for feline bartonellosis [66].

The current study used primers targeting the *gltA* gene of *Bartonella* spp. for screening. Using *gltA* detection, this gene had a high specificity for *Bartonella* detection in extracted DNA samples from blood [67]. The current BLAST results for the *gltA* and r*ibC*-amplified amplicons revealed a similar species of *Bartonella*. Additionally, other studies mentioned that positive *Bartonella* samples targeting the *ribC* gene were also positive to *gltA* in all samples due to the higher detection power of *gltA* [68,69,70]. However, there was no correlation for the BLAST results with the other housekeeping genes (*rpoB*, *ftsZ*, and *groEL*) in the current study. This might have been due to the low discriminatory power of some genes, except for *rpoB*, for species differentiation in the *Bartonella* genus [67]. On the other hand, the lack of correlated BLAST results might have been due to the co-infection of *Bartonella* spp. in individual cats. From the results, it was difficult to make any conclusions regarding the mixed infection at this time. Nevertheless, co-infection should be investigated using other more sensitive techniques.

*Bartonella*-positive free-ranging cats had raised values for MCV. Over 36% of bacteremic cats had low values for HGB and MCHC. Another study in dogs mentioned that *Bartonella*-infected blood was significantly low in hemoglobin (HGB), erythrocytic mass (RBC), and hematocrit (HCT) [71]. The same trend was reported in a hematological study in camels, where the *Bartonella*-positive animals were significantly low in HGB, MCH, and MCHC [72]. The mechanisms of anemia were studied mainly in *B. bacilliformis* and were described in various ways [73]. *Bartonella*-positive animals frequently showed anemia, eosinophilia, neutrophilia, and thrombocytopenia in [14,74]. Additionally, *Bartonella*-infected cats mostly showed mild or no abnormality of blood panels [18]. However, one hematological study in domestic cats revealed that MCV was noticeably low [75]. Interestingly, the hematological abnormalities caused by *Bartonella* spp. were rarely described in naturally infected cats presented as healthy carriers [11,76]. For significantly raised MCV values, we suggested that its volume was increased from two main ways: (1) From multiplied daughter cells of *Bartonella* in red blood cells in the replication stage [11], and (2) from another previous infection or immune-mediated disorder [28]. In addition, strong evidence suggested that heme compounds were necessary for *B. henselae* growth and hemoglobin was a potential source of heme in vivo [77] which explained the low values for HGB and MCHC in over 36% of *Bartonella*-positive cats in this study. Nevertheless, there are some limitations including the criteria for owned animals’ inclusion. A significant inclusion criterion for blood donors of studied samples that might affect *Bartonella* detection in owned animals required an ectoparasite control. Flea- and tick-controlled animals were possibly a chance of negative *Bartonella* detection. Due to the ectoparasite infestation, there was a risk factor for *Bartonella* infection in dogs and cats [78]. However, *Bartonella* detection in ectoparasite was not included in this study. For further study, *Bartonella* spp. in animal-infesting ectoparasites should be examined to describe the dynamics of pathogen transmission.

## 4. Materials and Methods

### 4.1. Animal Ethics Consideration

Blood samples were collected by veterinarians and involved the gentle restraint technique. This animal research study was approved by the Kasetsart University Institutional Animal Care and Use Committee under the Ethical Review Board of the Office of National Research Council of Thailand (NRCT). All the laboratories used in this study met the standards followed by verification of the Institutional Biosafety Committee (IBC), Faculty of Veterinary Medicine, Kasetsart University (approval ID ACKU63-VET-048).

### 4.2. Definition of Surveyed Population

In this study, we surveyed both free-ranging and owned animals. Free-ranging animals were dogs and cats residing in temples of Bangkok. These animals were fed by animal care takers of each temple. Moreover, the ectoparasite control history of free-ranging animals was unknown. Owned animals were dogs and cats registered at the Blood Bank (BB) unit of Kasetsart University Veterinary Teaching Hospital (KUVTH) for blood donors. Owned animals lived only in the household and they roamed only in the area around their houses. The criteria for blood donors at BB of KUVTH included a limited age (1–7 years), flea and tick continue control, no blood receive history, no drug usage, and required body weight (dogs ≥ 17 kg and cats ≥ 4 kg).

### 4.3. Study Sites and Sample Collection

This cross-sectional study was conducted in the Bangkok metropolis of Thailand. The animal caretakers and owners of animals that resided in temples and registered with the BB at KUVTH, respectively were invited to participate in this study. Animal that had resided in temples were defined as free-ranging animals and animals that had donated blood at the BB of KUVTH were defined as owned animals. The temples that consented in this study were in three zones of Bangkok (Figure 1). Thirty-four districts were intervened, and the number of animals per temples were based on the difficulty of handling and density of animals in the area. The data of the studied population are presented in Supplementary Table S4. Blood samples from the free-ranging dogs were collected from the cephalic or saphenous vein depending on the size of the animal and in the donor dog samples were collected from the jugular vein. Additionally, cat samples consisted of punctured venous blood only from the jugular vein in both free-ranging and donor cats. Approximately 3 mL of blood was placed into a sterile blood collection tube with EDTA anticoagulant. Two hundred microliters of collected blood was separated for molecular detection of *Bartonella* spp. and the remaining volume was used for hematological evaluation.

### 4.4. Animal’s General Data Collection

General data were collected for both free-ranging and owned animals including gender, living area, breed, age, and ectoparasite presentation at the surveyed time. Data of free-ranging animals were interviewed from the animal caretaker, however, data of owned animals were brought from the KUVTH record database and BB unit logbook. For ectoparasite presentation, they were examined at the time of blood collection and type of ectoparasite was recorded.

### 4.5. Genomic DNA Extraction

The separated blood samples were extracted for genomic DNA using commercial kits (FavorPrep^TM^ Blood DNA Extraction Mini Kit, Favorgen Biotech Corporation, Pingtung, Taiwan) following the manufacturer’s instructions. The final volume of eluted solution (100 µL) was stored at −20 °C until the PCR-based detection.

### 4.6. PCR Quality Control

Each PCR reaction was conducted with negative and positive control samples. Nuclease-free water was used as the *Bartonella* negative control. *B. henselae* Houston-1 extracted DNA was provided from the National Institute of Health, Department of Medical Science, Ministry of Public Health, Thailand and used as the positive control sample.

### 4.7. Bartonella Screening Using PCR

Conventional PCR was performed to detect *Bartonella* spp. in the extracted samples. First, all of the samples were screened for *Bartonella* spp. using a primer set targeting a partial fragment of the citrate synthase (*gltA*) gene (Table 5). The amplification conditions were controlled by a thermocycler (Mastercycler^®^ nexus gradient, Eppendorf, Hamburg, Germany). The 25 µL of PCR mixture contained 0.5 µL of dNTPs solution (0.2 mM each), 1X of Taq Reaction buffer (mixed with MgSO_4_), 4 pmol/µL of each primer, 0.04 U/µL of Taq DNA polymerase (Taq DNA Polymerase, Applied Biological Materials (ABM^®^) Inc., Richmond, BC, Canada), 0.8% of dimethyl sulfoxide, and 3 µL of DNA template. The amplified products were kept at 4 °C until electrophoresis. Electrophoresis was conducted using agarose gel under a 0.5X TAE buffer. The process of electrophoresis was run at 100 V for 40–60 min depending on the expected amplicon size. The sample bands which were suspected as being positive for the *Bartonella* amplicon size were purified using a DNA purification kit (Gel and PCR Purification System, BioFACT™, Daejeon, South Korea), according to the manufacturer’s instructions.

### 4.8. Other Housekeeping Gene Amplification

The *Bartonella*-suspected samples were amplified for other housekeeping gene fragments (Table 5) consisting of the beta-subunit of RNA polymerase (*rpoB*), cell division protein (*ftsZ*), 60 kDa chaperonin (*groEL*), and riboflavin synthase (*ribC*). The amplification cycles for each housekeeping gene are shown in Table 6. Additionally, the PCR mixture, amplified amplicon, gel electrophoresis, and DNA purification were used as for the above description of *gltA*.

### 4.9. DNA Sequencing and Phylogenetic Analyses

The purified amplicons were analyzed using Sanger’s sequencing technology by a commercial company (Macrogen^®^, Seoul, Korea). The obtained DNA sequences were edited using the SnapGene ^®^ Viewer software version 5.2.4 (https://www.snapgene.com/snapgene-viewer, accessed on 20 March 2021) and the phylogenetic relationship was analyzed using the MEGA-X software (https://www.megasoftware.net, accessed on 20 March 2021). Concatenated sequences were used to establish a phylogenetic tree under the fitted parameter model of the maximum likelihood method with 500 bootstrapping replications. All amplicon sequences were submitted to GenBank (accession numbers MW575344–MW575394).

### 4.10. Hematological Analyses

The remaining blood samples were sent to the Hematological Unit of KUVTH and to a private hematological company. All of the blood samples were analyzed using the laser flow cytometry technique by an automated hematology analyzer (Sysmex XT-2000*i*V™, IDEXX Bioresearch, Norderstedt, Germany). Additionally, a manual differential count from the Diff-Quick stained blood smear was also performed in all of the samples. Common blood panels were analyzed based on the leukocyte count (WBC) and the proportions of neutrophils (NEU), lymphocytes (LYM), eosinophils (EOS), monocytes (MON), and by the numbers of basophils (BAS), the erythrocytic count (RBC), hemoglobin concentration (HGB), packed cell volume (PCV), mean corpuscular volume (MCV), mean corpuscular hemoglobin (MCH), mean corpuscular hemoglobin concentration (MCHC), and platelet count (PLT).

### 4.11. Statistical Analyses

General data on the tested animals were recorded for age, sex, breed, roaming/living location, ectoparasitic infestation, and living pattern. The general and hematological data were statistically analyzed using the R programming language version 4.0.2. Variables of interest were tested as risk factors using Chi square or Fisher’s exact test depending on the nature of the recorded data. Only the significant factors were analyzed, and a multiple comparison used the Bonferroni correction method and calculated odds ratio. A comparison of hematological values was tested between *Bartonella*-positive and *Bartonella*-negative groups using the Mann-Whitney U test. All statistical analyses were processed using 95% confidence intervals and a *p*-value < 0.05 was considered as significant.

## 5. Conclusions

Overall, the prevalence of *Bartonella* spp. in domestic animals in Bangkok, Thailand was 1.61% and was only identified in free-ranging cats (2.83%). There was no evidence of *Bartonella* in domestic dogs. Two zoonotic species were identified in this study (*B. henselae* and *B. clarridgeiae*). There was a high proportion detected in temple clusters located in the inner zone of Bangkok, however, there were no significant differences for other associated factors. The 100% bootstrapping replication of the concatenated phylogenetic tree based on the *gltA* and *ribC* genes confirmed the two species of *Bartonella* spp. in this study. Nevertheless, other genes (*rpoB*, *ftsZ*, and *groEL*) revealed different species from the same individual sample and mixed infection was expected. *Bartonella*-positive, free-ranging cats had raised MCV values compared to negative cats, additionally, there were low values of HGB and MCHC in over 36% of the *Bartonella*-positive cats. Based on these results, we suggest that zoonotic *Bartonella* spp., especially *B. henselae* and *B. clarridgeiae*, are important in Thailand and a prevention program, such as routine health checks of animals and humans, should be implemented. Importantly, abnormal red blood cell parameters such as a high corpuscular volume or low hemoglobin-related parameters in healthy cats should also be considered as indicators of feline subclinical bartonellosis in Thailand. Moreover, the knowledge of *Bartonella*-related diseases protection should be intervened in the risk person.

## Figures and Tables

**Table 1:** Number of related factors of examined animals in this study.

Factors	Free Ranging		Owned	
	Dog	Cat	Dog	Cat
Gender ^1^				
Male	82	206	64	32
Female	92	254	57	19
Studied zone				
Inner	65	223	-	-
Intermediate	48	169	-	-
Outer	61	68	-	-
Breed				
Purebred	0	0	90	6
Crossbred	174	460	31	47
Age group				
<1 year	42	66	0	0
1–5 years	100	362	97	47
>5 years	32	32	24	6
Ectoparasites				
Yes	16	223	0	0
- Flea	1	223	0	0
- Tick	9	0	0	0
- Flea and tick	6	0	0	0
No	158	237	121	53
Total	174	460	121	53

^1^ Gender of two owned cats were unrecorded.

**Table 2:** Number and prevalence of *Bartonella* spp. with 95% confidence interval of studied dogs and cats in this study.

Host	No. Positive/No. Tested	% *Bartonella* Positive	95% Confidence Interval
Free ranging	0/174	0%	-
Owned	0/121	0%	-
Total dogs	0/295	0%	-
Free ranging	13/460	2.83%	1.51–4.78%
Owned	0/53	0%	-
Total cats	13/513	2.53%	1.36–4.29%

**Table 3:** Accession number of each sequence submitted to the GenBank database, BLAST results, and identity percentage.

ID	*gltA*		*rpoB*		*ftsZ*		*groEL*		*ribC*	
	ACNO	BLAST	ACNO	BLAST	ACNO	BLAST	ACNO	BLAST	ACNO	BLAST
02901	MW575345	Bc	MW575391	Bh	MW575370	02901	MW575345	Bc	MW575391	Bh
	[94.59–100%]		[99.41–100%]		[99.23–100%]		[98.94–100%]		[99.54–100%]	
03302	MW575346	Bc	MW575386	Bh	MW575371	03302	MW575346	Bc	MW575386	Bh
	[94.59–100%]		[99.41–100%]		[99.23–100%]		[98.94–100%]		[99.54–100%]	
04102	MW575347	Bc	(-)		MW575372	04102	MW575347	Bc	(-)	
	[94.59–100%]				[99.23–100%]				[94.59–100%]	
05909	MW575348	Bc	MW575392	Bh	MW575373	05909	MW575348	Bc	MW575392	Bh
	[94.59–100%]		[99.41–100%]		[99.23–100%]		[98.94–100%]		[99.54–100%]	
06806	MW575349	Bc	MW575390	Bh	MW575379	06806	MW575349	Bc	MW575390	Bh
	[94.59–100%]		[98.95–100%]		[99.39–100%]		[98.94–100%]		[99.54–100%]	
07406	MW575350	Bc	(-)		(-)	07406	MW575350	Bc	(-)	
	[94.59–100%]							[94.59–100%]		
08303	MW575351	Bc	(-)		(-)	08303	MW575351	Bc	(-)	
	[94.59–100%]								[94.59–100%]	
08501	MW575353	Bh	MW575387	Bh	MW575374	08501	MW575353	Bh	MW575387	Bh
	[97.01–100%]		[99.41–100%]		[99.23–100%]					
08508	MW575352	Bc	(-)		(-)	08508	MW575352	Bc	(-)	
	[94.59–100%]								[94.59–100%]	
09304	MW575354	Bh	MW575388	Bh	MW575375	09304	MW575354	Bh	MW575388	Bh
	[97.01–100%]		[99.41–100%]		[99.23–100%]					
09306	MW575344	Bh	MW575393	Bh	MW575376	09306	MW575344	Bh	MW575393	Bh
	[97.01–100%]		[99.41–100%]		[99.23–100%]					
09701	MW575355	Bh	MW575389	Bh	MW575377	09701	MW575355	Bh	MW575389	Bh
	[97.01–100%]		[99.41–100%]		[99.23–100%]					
09707	MW575356	Bh	MW575394	Bh	MW575378	09707	MW575356	Bh	MW575394	Bh
	[97.01–100%]		[99.41–100%]		[99.23–100%]					

ACNO: Accession number; Bc: *Bartonella clarridgeiae*; Bh: *Bartonella henselae*; (-): Amplicon absence; [ ]: % identity.

**Table 4:** Number of examined dogs and cats classified by hematological values *.

**Parameter**	**Owned Dogs (*n* = 122)**			**Owned Cats (*n* = 52)**		
	**Low**	**Normal**	**High**	**Low**	**Normal**	**High**
PCV	1 (0)	121 (0)	-	2 (0)	36 (0)	14 (0)
(%)	[33.10]	[47.23 ± 3.71]		[32.05 ± 3.75]	[41.03 ± 2.29]	[47.33 ± 1.60]
RBC	-	113 (0)	9 (0)	-	49 (0)	3 (0)
(×10^6^/µL)		[6.84 ± 0.55]	[8.17 ± 0.40]		[8.71 ± 0.72]	[10.48 ± 0.11]
HGB	-	117 (0)	5 (0)	1 (0)	45 (0)	6 (0)
(g/dL)		[16.28 ± 1.14]	[19.26 ± 0.23]	[9.14]	[13.73 ± 0.90]	[16.03 ± 0.36]
MCV	24 (0)	98 (0)	-	-	51 (0)	1 (0)
(fL)	[62.83 ± 2.44]	[69.33 ± 1.84]			[47.81 ± 3.20]	[18.37]
MCH	2 (0)	118 (0)	2 (0)	-	48 (0)	4 (0)
(pg)	[20.99 ± 0.00]	[23.70 ± 1.13]	[26.69 ± 0.41]		[15.64 ± 0.82]	[17.59 ± 0.52]
MCHC	-	112 (0)	10 (0)	-	48 (0)	4 (0)
(g/dL)		[34.68 ± 0.92]	[37.15 ± 0.91]		[32.64 ± 0.93]	[36.71 ± 0.21]
PLT	39 (0)	83 (0)	-	16 (0)	36 (0)	-
(×10^3^/µL)	[187.10 ± 29.76]	[267.41 ± 47.82]		[221.32 ± 51.54]	[361.11 ± 50.75]	
WBC	-	106 (0)	16 (0)	-	51 (0)	1 (0)
(×10^3^/µL)		[10.34 ± 2.32]	[15.14 ± 0.72]		[10.88 ± 3.34]	[19.80]
NEU	11 (0)	105 (0)	6 (0)	2 (0)	26 (0)	24 (0)
(%)	[54.27 ± 4.58]	[72.20 ± 6.91]	[89.00 ± 2.53]	[37.85 ± 4.03]	[56.35 ± 4.52]	[75.58 ± 8.53]
LYM	6 (0)	78 (0)	38 (0)	26 (0)	18 (0)	9 (0)
(%)	[4.67 ± 1.97]	[15.96 ± 3.74]	[29.18 ± 5.65]	[17.42 ± 6.10]	[30.65 ± 3.17]	[42.46 ± 5.20]
EOS	-	113 (0)	9 (0)	-	20 (0)	32 (0)
(%)		[4.18 ± 2.52]	[12.43 ± 3.51]		[2.34 ± 1.42]	[7.29 ± 2.57]
MON	35 (0)	86 (0)	1 (0)	-	31 (0)	21 (0)
(%)	[0.31 ± 0.30]	[5.21 ± 2.20]	[11.00]		[1.58 ± 1.34]	[7.67 ± 1.59]
BAS	-	122 (0)	-	-	52 (0)	-
(%)		[0.04 ± 0.03]			[0.11 ± 0.10]	
**Parameter**	**Free-Ranging Dogs (*n* = 133)**			Cats (*n* = 343) **		
	**Low**	**Normal**	**High**	**Low**	**Normal**	**High**
PCV	47 (0)	86 (0)	-	147 (4)	167 (6)	29 (1)
(%)	[29.09 ± 5.13]	[43.68 ± 4.89]		[30.30 ± 4.04]	[39.14 ± 2.64]	[47.31 ± 2.01]
RBC	55 (0)	74 (0)	4 (0)	16 (2)	318 (9)	9 (0)
(×10^6^/µL)	[3.96 ± 0.77]	[6.14 ± 0.76]	[8.29 ± 0.37]	[4.18 ± 0.81]	[7.53 ± 1.11]	[10.49 ± 0.54]
HGB	70 (0)	60 (0)	3 (0)	69 (4)	271 (7)	3 (0)
(g/dL)	[9.26 ± 1.95]	[14.11 ± 1.51]	[19.30 ± 0.35]	[8.45 ± 1.19]	[12.12 ± 1.39]	[16.50 ± 0.78]
MCV	14 (0)	83 (0)	36 (0)	2 (0)	308 (9)	33 (2)
(fL)	[60.79 ± 3.66]	[72.08 ± 2.87]	[82.62 ± 4.38]	[35.35 ± 0.49]	[47.80 ± 3.48]	[58.15 ± 3.95]
MCH	27 (0)	105 (0)	1 (0)	4 (0)	317 (10)	22 (1)
(pg)	[19.57 ± 1.53]	[22.62 ± 1.12]	[30.50]	[12.33 ± 0.49]	[15.27 ± 0.87]	[17.78 ± 1.06]
MCHC	112 (0)	20 (0)	1 (0)	72 (5)	264 (6)	7 (0)
(g/dL)	[29.32 ± 1.74]	[33.49 ± 1.00]	[36.50]	[28.84 ± 0.81]	[32.37 ± 1.43]	[36.83 ± 0.64]
PLT	112 (0)	21 (0)	-	82 (3)	256 (8)	5 (0)
(×10^3^/µL)	[101.36 ± 46.74]	[312.10 ± 93.05]		[230.82 ± 56.01]	[461.95 ± 116.42]	[870.00 ± 53.46]
WBC	4 (0)	69 (0)	60 (0)	3 (1)	210 (8)	130 (2)
(×10^3^/µL)	[4.01 ± 0.89]	[10.40 ± 2.53]	[19.49 ± 4.63]	[5.32 ± 0.16]	[14.83 ± 2.94]	[26.57 ± 7.67]
NEU	69 (0)	61 (0)	3 (0)	60 (2)	184 (5)	99 (4)
(%)	[46.71 ± 8.73]	[66.85 ± 6.24]	[87.17 ± 2.65]	[37.47 ± 6.36]	[54.84 ± 5.34]	[70.61 ± 5.78]
LYM	4 (0)	40 (0)	89 (0)	116 (3)	100 (5)	127 (3)
(%)	[5.33 ± 1.77]	[16.13 ± 3.41]	[35.77 ± 12.15]	[20.36 ± 4.82]	[30.95 ± 2.69]	[47.40 ± 24.71]
EOS	-	86 (0)	47 (0)	-	74 (3)	269 (8)
(%)		[4.87 ± 2.34]	[13.97 ± 4.13]		[2.48 ± 1.40]	[9.10 ± 4.30]
MON	8 (0)	110 (0)	15 (0)	-	306 (9)	37 (2)
(%)	[0.21 ± 0.20]	[5.57 ± 2.22]	[11.17 ± 1.15]		[2.47 ± 1.29]	[6.61 ± 2.08]
BAS	0	133 (0)	0	-	343 (11)	-
(%)	-	[0.22 ± 0.18]	-		[0.12 ± 0.10]	

* Numbers in ( ) are the *Bartonella*-positive sample in each hematological class; [ ] is the average value of each hematological value. ** There were 11 of 13 *Bartonella* infected free-ranging cats that obtained blood parameters.

**Table 5:** Primer list used for *Bartonella* detection in this study.

Primer Name	Gene	Direction	Primer Sequence	Amplicon Size (bp)	Reference
BhCS.781p	*gltA*	Forward	GGGGACCAGCTCATGGTGG	379	[68]
BhCS.1137n		Reverse	AATGCAAAAAGAACAGTAAACA		
1400F	*rpoB*	Forward	CGCATTGGCTTACTTCGTATG	795	[79]
1400F		Reverse	GTAGACTGATTAGAACGCTG		
BaftsZF	*ftsZ*	Forward	GCTAATCGTATTCGCGAAGAA	885	[80]
BaftsZR		Reverse	GCTGGTATTTCCAAYTGATCT		
HSPF1d	*groEL*	Forward	GAACTNGAAGATAAGTTNGAA	1188	[81]
BbHS1630.n		Reverse	AATCCATTCCGCCCATTC		
BARTON-1	*ribC*	Forward	TAACCGATATTGGTTGTGTTGAAG	540	[69]
BARTON-2		Reverse	TAAAGCTAGAAAGTCTGGCAACATAACG		

bp: Base pair.

**Table 6:** Polymerase chain reaction conditions of each housekeeping gene for *Bartonella* detection.

Gene	Initial Denaturation	Denaturation	Annealing	Extension	Final Extension	Repeated Cycle
*gltA*	95 °C (5 m)	95 °C (20 s)	51 °C (30 s)	72 °C (2 m)	72 °C (5 m)	35
*rpoB*	94 °C (2 m)	94 °C (30 s)	53 °C (30 s)	72 °C (1 m)	72 °C (2 m)	35
*ftsZ*	94 °C (4 m)	94 °C (30 s)	55 °C (30 s)	68 °C (1 m)	68 °C (10 m)	44
*groEL*	94 °C (3 m)	94 °C (30 s)	54 °C (30 s)	72 °C (1.5 m)	72 °C (7 m)	40
*ribC*	95 °C (10 m)	95 °C (1 m)	51 °C (1 m)	72 °C (1 m)	72 °C (3 m)	37

m: Minute(s).

## Data Availability

Data is contained within the article and supplementary materials.

## Supplementary Materials

The following are available online at https://www.mdpi.com/article/10.3390/pathogens10050503/s1. Table S1: Details of sequencing data obtained in this study and their related-BLAST; Table S2: The reference of blood parameters used for cut-off in this study; Table S3: Comparison of positive and negative-*Bartonella* proportions between a recent investigation and other reports discussed in this study; Table S4: The data of studied population in this study.
